# The characteristics of PM emissions from construction sites during the earthwork and foundation stages: an empirical study evidence

**DOI:** 10.1007/s11356-023-26494-4

**Published:** 2023-03-22

**Authors:** Hui Yan, Qiqi Li, Kailun Feng, Lei Zhang

**Affiliations:** 1 Department of Construction Management, South China University of Technology, Guangzhou, 510641 China; 2 Department of Applied Physics and Electronics, Umeå University, 901 87 Umeå, Sweden; 3 Department of Construction Management, Guangzhou University, Guangzhou, 510006 China

**Keywords:** Construction site, Earthwork and foundation stage, PM emission, Construction activity, Reduction measure, Environmental impact

## Abstract

The bulk of the particulate matter (PM) emissions generated during construction projects are significantly released during the earthwork and foundation stages. To reduce and control these emissions, it is necessary to have reliable data on their characteristics. However, construction PM are poorly characterized because their composition depends on several factors (e.g., weather and reduction measures) and various on-site activities whose effects may interact. To address these challenges, a long-term quantitative empirical study using advanced statistical methods was performed on a real construction project during the whole earthwork and foundation stages. The upwind-downwind method was used to collect data on PM emissions throughout the earthwork and foundation construction process, and correlation analysis, paired samples *t*-test, and partial least squares regression (PLS) were used to analyze TSP, PM_10_, and PM_2.5_ emissions and their relationships with various influencing factors. The results showed that both earthwork and foundation constructions generate substantial PM emissions because there were differences with statistical significances in the PM levels measured upwind and downwind of the construction site. TSP and PM_10_ emissions correlated moderately with humidity and wind speed. However, temperature and atmospheric pressure did not correlate significantly with any of the measured emissions. The main activities responsible for PM emissions during the earthwork and foundation construction stages were hammer piling, waste stacking, and materials transportation. Water spraying was found to effectively reduce TSP and PM_10_ emissions, while the use of a fog cannon more effectively reduced PM_2.5_ emissions. Construction PM is an important source of atmospheric pollution in cities; the findings presented herein provide cornerstone and knowledge to guide efforts for reducing its impact.

## Introduction

Construction work threatens the ecological environment and human health in many rapidly urbanizing regions because it generates many pollutants, including solid waste, sound pollution, greenhouse gas emissions, and particulate matter (PM). Construction PM consists of solid and liquid particles suspended in the air (USEPA 1996), that are discharged from equipment and materials used in various stages of construction and as a result of construction activities. These particles do not only affect the construction site; they are carried by air flows and diffusion into surrounding areas. Construction PM therefore contributes significantly to the overall burden of urban atmospheric pollution (Guttikunda and Calori 2013; Faber et al. 2015). The emission and diffusion of construction PM depends on the construction activities that are performed, the applied PM reduction measures, the weather, project-specific factors, the type of PM under consideration, and the randomness of PM diffusion. Because of this complexity, little is known about the characteristics of construction PM, the activities and processes primarily responsible for its generation, or ways of effectively controlling its emissions.

Large quantities of PM with different aerodynamic equivalent diameters are generated during the four main stages of building construction, namely the earthwork, foundation, main structure, and decoration stages (Araújo et al. 2014). Three main size-based categories of PM have been defined: TSP (aerodynamic equivalent diameter ≤ 100μm), PM_10_ (aerodynamic equivalent diameter ≤ 10μm), and PM_2.5_ (aerodynamic equivalent diameter ≤ 2.5μm). All three categories cause serious pollution of the atmospheric environment of the construction site and its surroundings. Both on-site workers and residents of the surrounding areas may suffer adverse short- and long-term health effects due to PM exposure (WHO 2006). Previous studies have shown that PM exposure is closely related to the occurrence of cardiovascular, respiratory, and skin diseases (Dockery 1994; Ngoc et al. 2017) and to increased mortality from various diseases (Dai et al. 2014)

Because of the health risks posed by PM and other airborne pollutants, several air quality standards have been issued to protect the atmospheric environment and human health. In 2005, the World Health Organization (WHO 2006) recommended that the annual average concentration and 24-h average concentration of PM10 should not exceed 20μg/m^3^ and 50μg/m^3^, respectively; the corresponding recommended limits for PM2.5 are 10μg/m^3^ and 25μg/m^3^. In 2012, the US National Ambient Air Quality Standard (NAAQS) (USEPA 2013) was revised for the fourth time, improving the standard limits of the concentration of PM2.5 and PM10. Previous studies have provided deep insights of the adverse health effects of construction PM on workers and surrounding residents (Yan et al. 2020).

To effectively control construction PM emissions, it is important to characterize the PM emissions generated during different stages of construction and identify ways of reducing them. Majority of construction PM emissions is originated from the earthwork and foundation construction stages (Fan et al. 2020). Therefore, efforts have been made to characterize the PM generated at these stages. However, the contributions of individual activities during the earthwork and foundation construction stages to overall emissions of different PM types remain unclear, and the effectiveness of various measures at reducing emissions of different types of construction PM is unknown. This makes it hard to suggest effective measures for reducing construction PM emissions during the earthwork and foundation stages. There is thus a need for a detailed study of PM emissions during these stages. In addition, these is a need to clarify the individual and combined effects of various factors that may influence construction PM emissions including the construction activities that are performed, the applied reduction measures, and the meteorological conditions.

Therefore, this paper presents a long-term empirical field study on the earthwork and foundation construction stages of a real construction project that was designed to address the knowledge gaps mentioned above. Meteorological parameters were recorded and concentrations of TSP, PM_10_, and PM_2.5_ were monitored in real time over three months. Descriptive statistics, correlation analysis, and partial least squares (PLS) regression were then used to investigate the correlations between PM emissions and meteorological parameters, construction activities, and PM reduction measures. The results obtained will provide valuable guidance in future efforts to design effective PM reduction measures for construction sites during the earthwork and foundation stages.

The structure of this paper is as follows. After this introduction chapter, section 2 outlines what is known about the factors influencing construction PM emissions and highlights some limitations of the existing literature. Section 3 explains the methods used for data collection and analysis. Section 4 analyzes the gathered empirical data, identifies key influencing factors, decomposes PM emissions from different construction activities, and quantifies the effectiveness of different reduction measures. Finally, section 5 summarizes the conclusions, contributions, and limitations of this work and offers suggestions for future research in this area.

## Literature review

### Factors influencing PM emissions

Construction activities are the main source of construction PM, but the PM emissions profiles of individual construction activities can differ markedly. Fan et al. (2020) found that PM concentrations vary widely depending on the stage of construction, the site size, and the nature of the construction project. In particular, PM concentrations were higher during the earthwork stage, at small construction sites, and during building engineering work than in other stages, at large construction sites, and during municipal engineering work, respectively. Separately, Zhao et al. (2010) estimated the PM_10_ emission factors for foundation excavation, foundation construction, earthwork backfilling (coarse graded laying) and general construction based on FDM simulation correction, and found significant gaps in PM emissions between different individual construction activities. It can be found that in previous studies, the emission differences of individual construction activities have been studied, but the correlation between pollution emissions and the concentration of PM in the atmospheric environment has not been fully discussed. In addition, PM emissions were found to vary with the average weight (Zhao et al. 2009), number (Raile 1996), and average speed (Cowherd et al. 1974) of the vehicles used on-site.

Meteorological conditions also strongly affect PM emissions and diffusion. Zhang et al. (2008) found that PM generation rate decreases significantly with the increase of air humidity and mass flow. When monitoring construction sites in Brazil, Moraes et al. (2016) discovered that the measured concentrations of TSP and PM10 increased markedly when the direction of the monitoring point from the construction site was aligned with the wind direction or the level of rainfall was low. Ruan et al. (2019) found that variation in atmospheric stability and the height of the emission source strongly affected the concentration distribution of PM: PM diffusion increased as atmospheric stability decreased and the ground concentration of PM was inversely proportional to the height of the emission source. Ge (2018) found that the correlations between PM concentrations and individual meteorological factors were weak but the combined effects of multiple meteorological elements strongly affected PM emissions. Other factors found to significantly affect PM emissions include soil moisture (Faber et al. 2015), soil silt content (Lee et al. 2001), surface moisture content, total road surface dust loading, and the dusting area rate (Zhao et al. 2010).

Finally, studies on control measures have shown that construction PM emissions can be reduced by spraying water to increase ground or soil humidity (Fitz and Bumiller 2000), sprinkling to increase air humidity (Zou 2021), reducing the dust by covering or using a dust suppressant (Yan et al. 2022; Luo et al. 2022), restricting traffic (Tian et al. 2009), and reducing air flow by enclosure (Zhao et al. 2021). However, the above conclusions are mostly based on the calculation of the transient dust removal efficiency, without monitoring the actual dust removal performance.

### Characteristics of construction PM emissions

Many previous studies have investigated the characteristics of construction PM emissions in terms of the diffusion space. Lin et al. (2018) measured PM emissions at 11 construction sites in the Pearl River Delta and found that the main areas with high PM concentrations during the monitoring period were construction roads, interiors under decoration, and building material stacking yards. Fan et al. (2020) found that construction PM concentrations initially increased markedly in the downwind direction but then declined and stabilized at about 50m. Tian et al. (2008) monitored the vertical and horizontal concentration distributions of dust fall (DF) at a construction site and found that equilibrium was reached at distances of 4m and 100m. Finally, Zhao et al. (2010) found that the decline ranges of TSP and PM_10_ concentrations in horizontal direction were higher than that of PM_2.5_ and PM_1_ in their study of municipal engineering.

There is also evidence that construction PM emissions are time-dependent. Xue et al. (2017) estimated construction PM emissions in Beijing from 2000 to 2015 using the emission factor method. And they concluded that the emission intensities of construction dust in summer and autumn were greater than in spring and winter. Similarly, Fujitani et al. (2012) compared the particle size distribution and number concentrations of PM in the air during summer and winter. They concluded that the temperature affected the concentrations of PM in the form of atmospheric dilution ratio and evaporation rate of PM. In addition, they found that the attenuation of PM with diameters below 30 nm was higher in winter than in summer after correcting for the effect of atmospheric dilution ratio; this was attributed to the difference in particle volatility.

There is evidence of correlations between the concentrations of PM with different particle sizes. Guo et al. (2014) analyzed data from several cities in Xinjiang, China, revealing a good correlation between TSP and PM_10_, and found that this correlation was stronger during hot and dusty periods than during non-dusty weather. Similarly, Huang et al. (2014) observed strong correlations between TSP, PM_10_, and PM_2_ in Harbin. However, it should be noted that the emissions of PM with different particle sizes may show divergent trends because individual variables may have differing levels of influence on particles of different sizes. In particular, smaller particles are more likely to diffuse evenly in the air than large particles (Zhong et al. 2010). Accordingly, Yan et al. (2020) concluded that the downwind distances within which construction activities influence TSP, PM_10_ and PM_2.5_ concentrations were about 100m, 50m ~ 100m, and 20 m ~ 50 m, respectively, when the wind speed was below 1.5 m/s. Additionally, Azarmi and Kumar (2016) measured PM_10_, PM_2.5_, and PM_1_ concentrations during demolition and found that the number of coarse particles released exceeded that of smaller particles.

### Summary of research status

As shown by the above literature review, previous studies on construction PM have provided valuable insights into the characteristics of these emissions and the factors influencing them. However, there are still some notable gaps in the literature. Previous studies have largely focused on overall PM emissions at construction site level and have only measured PM emissions during specific construction activities. However, there is a need for long-term data representing PM emissions from multiple different construction activities across different phases of a construction project. In addition, previous studies have not evaluated the effectiveness of different PM reduction measures during individual construction activities. To address these limitations, this paper presents a long-term empirical study on PM emissions from a construction site during the entirety of the earthwork and foundation stages, characterizes the PM emissions associated with various construction activities, and evaluate the effectiveness of different PM reduction measures.

## Methods

**Fig. 1:**
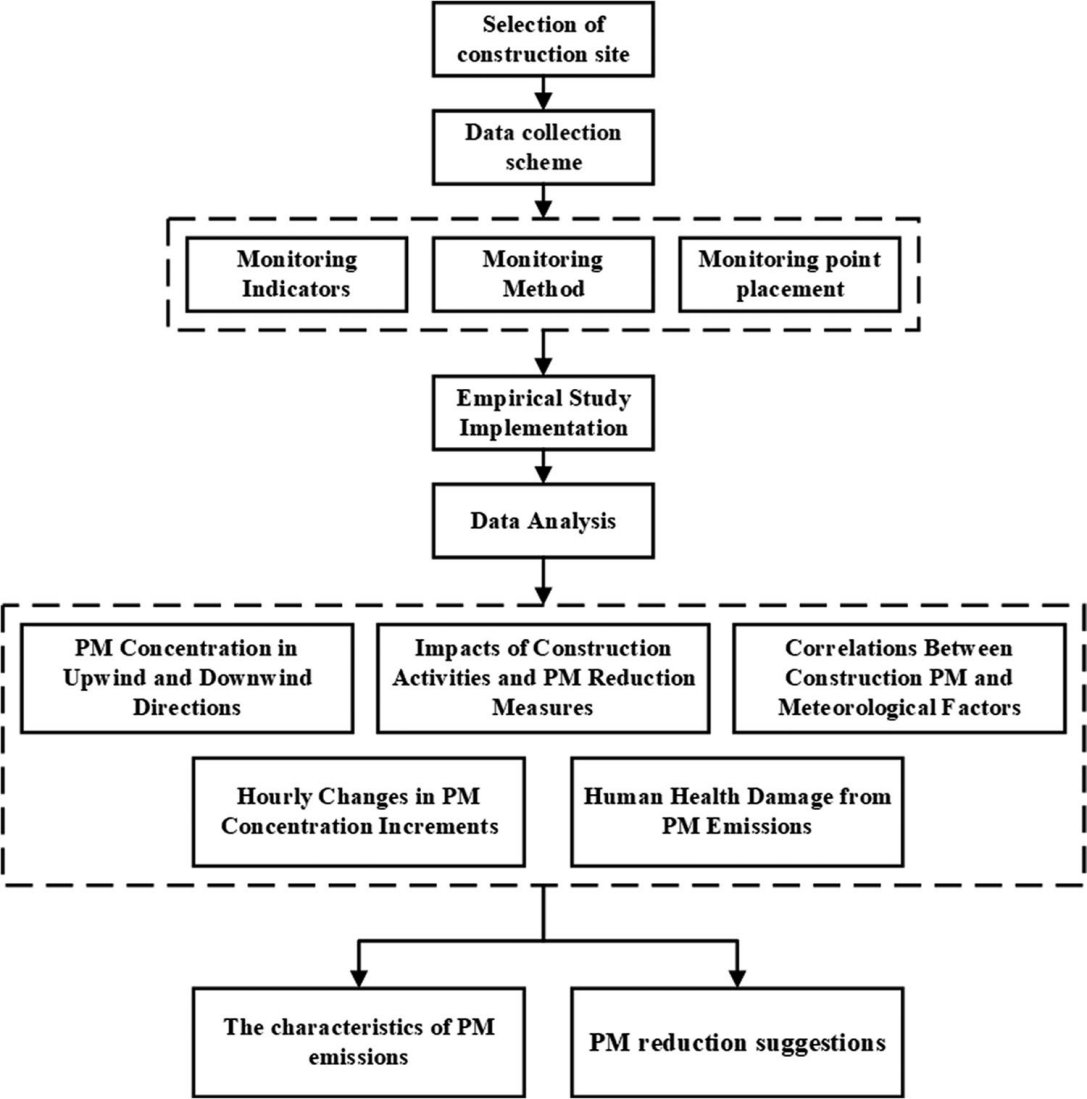
The overall framework of the empirical study

A typical construction project in a metropolitan area (Guangzhou, China) was selected as the empirical case. Empirical data were collected throughout the project’s earthwork and foundation construction stages. TSP, PM_10_, and PM_2.5_ concentrations were measured continuously together with the temperature, humidity, atmospheric pressure, and wind speed. In addition, the main construction activities being undertaken and the PM reduction measures in use were recorded. The significance of changes in PM concentrations over time was evaluated using paired samples t-tests, correlation analysis, and partial least squares (PLS) regression. As a result, the effects of individual construction activities, meteorological factors, and PM reduction measures on construction PM concentrations in areas adjacent to the construction site were evaluated. The following section outlines the data collection methods used in this work, describes the implementation of the empirical study, and explains the methods used for data analysis. The overall framework of the empirical study is shown in Fig. 1.

### Data collection

#### Monitoring indicators

In some countries, China for example, the standard specifies limits on construction PM emissions based on TSP and PM_10_ concentrations. However, general air quality standards typically use PM_10_ and PM_2.5_ as indicators of atmospheric particulate pollution. A large proportion of PM_2.5_ may consist of secondary aerosols generated by secondary reactions of gaseous and particulate substances in the air (Huang et al. 2014). Therefore, although PM_10_ and PM_2.5_ concentrations in the air often exhibit similar trends, these indicators are not interchangeable. Therefore, to comprehensively characterize construction PM emissions, concentrations of TSP, PM_10_ and PM_2.5_ were all measured in this empirical study.

#### Monitoring method

**Fig. 2:**
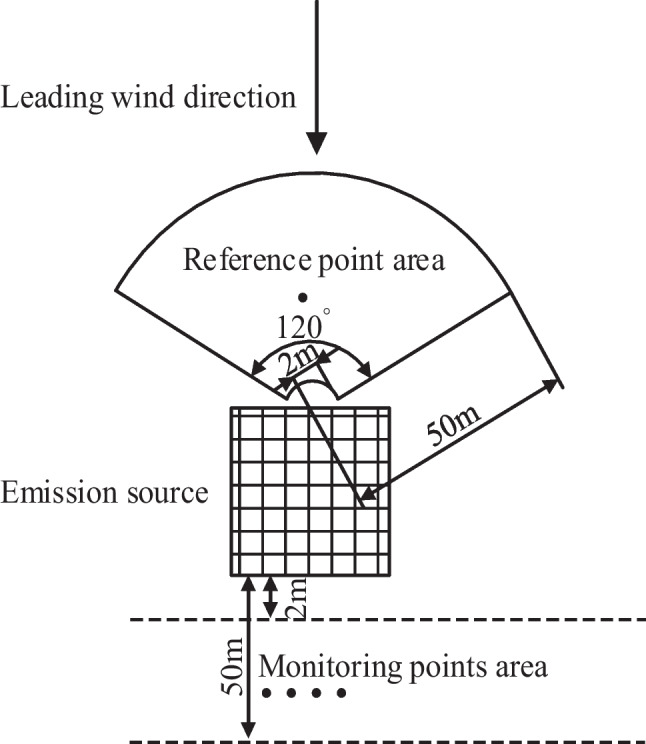
Upwind and downwind measuring point locations relative to the emissions source

Methods based on light scattering are commonly used to measure PM concentrations in air (Ministry of Labor of the People’s Republic of China 1996; Yan et al. 2020), because they enable real-time and long-term continuous monitoring. PM emissions during construction can be regarded as fugitive emissions. The standard for monitoring fugitive emissions stipulates that when monitoring such emissions one should first determine the dominant wind direction based on weather forecasts and an anemoscope, then apply the upwind-downwind method (Chinese HJ/T 55-2000). This approach was adapted in this study to monitor construction PM concentrations (see Fig. 2). HN-CK3000 Dust On-line Monitoring Systems were used to measure the concentrations of TSP, PM_10_ and PM_2.5_, together with data on the meteorological conditions at the construction site. The reference point (upwind of the construction site) was located between 2 and 50 m from the emission source in the upwind direction, in a 120° annular area as shown in Fig. 2. Additional downwind monitoring points were distributed between 2 and 50 m away from the emission source (Fig. 2). Reasonable locations for PM monitoring equipment were chosen by considering the properties of the construction site such as its topography and layout as well as safety factors and excluding irrelevant emissions.

The concentrations of TSP, PM_10_, and PM_2.5_ were continuously monitored at each monitoring point for 24 h per day, together with the temperature, humidity, atmospheric pressure, and wind speed. In addition, various construction activities and PM reduction measures at the construction site were recorded so that they could be related to the measured PM concentrations. The daily average PM_10_ and PM_2.5_ concentrations in the city where the project was located during the studied period were obtained from the national air quality monitoring points of the Air Quality Report System (http://www.cnemc.cn) and regarded as the background PM concentrations.

The maximum measured value of the concentration in the monitoring point is taken as the downwind dust concentration, and the difference between the downwind dust concentration and the upwind dust concentration is taken as the dust concentration increment, which represents the average emission level of particles in the construction site and reveals the influence of the construction site on the atmospheric environment outside the site. Considering that the construction site area is relatively small and the variation of meteorological parameters is small, the average temperature, humidity, atmospheric pressure and wind speed monitored by the three instruments are taken as the meteorological parameter values of the sample site.

### Empirical study implementation

The empirical case examined in this work is a construction site in Guangzhou, southern China. Guangzhou is China’s third largest city in terms of population and has a highly active construction sector. Construction projects in such a densely populated city could potentially affect the health of many residents of surrounding areas. The monitoring period and the project’s earthwork and foundation construction stages occurred during the dry season, when rainfall is comparatively low and the construction PM could diffuse easily. It also makes this construction project relatively straightforward to evaluate the contribution of construction PM to atmospheric pollution. The chosen project was therefore well suited to serve as the empirical case for this study.

**Fig. 3:**
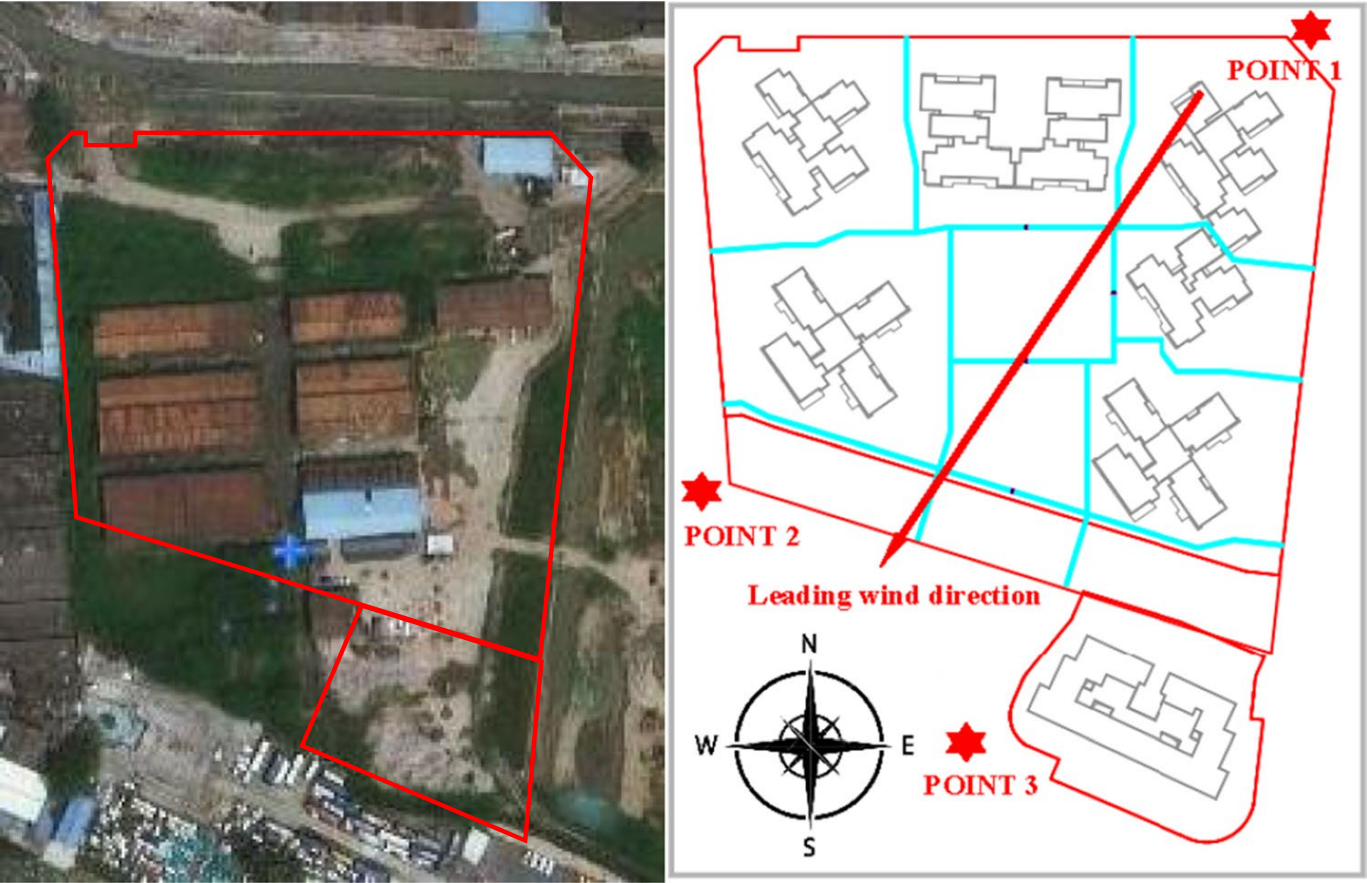
Aerial and plan views of the construction site and monitoring point locations

The neighborhood of the empirical case was also favorable for data collection as shown in Fig. 3: the case site is adjacent to a river to the north, the Pearl River to the south, an abandoned industrial park to the west, and a wasteland to the east. This is advantageous because it reduces the likelihood that the measured PM concentrations will be affected by irrelevant off-site emission sources.

The studied construction project comprises 7 residential buildings and 1 commercial office building. Each residential building has 38 ~ 39 floors above the ground and 2 ~ 3 floors underground, and the commercial building has 17 floors. The overall floor area is 216965 m^2^ and the construction site covers an area of 25791 m^2^. Table 1 provides more detailed information on the case site.

**Table 1:** Characteristics of the studied construction site

Type of project	Structure type	Scope of the work (Aboveground/Underground)	Area of land	The soil of land	Construction stage
Residential and commercial	Frame-shear wall structure	38(39)/3 stories for residential building; 17/2 stories for commercial office	25791 m^2^	Medium coarse sand	Earthwork and foundation

During the monitoring period, a range of earthwork and foundation construction activities were conducted at the site. The dominant wind was from the northeast. Three monitoring points were established inside the construction enclosure to monitor PM concentrations and meteorological data, including one upwind reference point (POINT-1) and two downwind monitoring points (POINT-2 and POINT-3). These monitoring points were chosen after considering the prevailing wind direction and safety conditions for the monitoring equipment and researchers. Each point was equipped with a HN-CK3000 Dust On-line Monitoring System with a monitoring height of 2.5 meters. The on-site data collection and field study lasted for a total of 84 days starting on October 11^th^, 2020 and ending on January 3^rd^, 2021.

### Data analysis

#### Treatment of monitoring data

The maximum concentration measured at the monitoring points (POINT-2 and POINT-3) was taken as the downwind PM concentration. The difference between the downwind PM concentration and the upwind PM concentration is referred to as the PM concentration increment and represents the PM emissions to the surrounding atmospheric environment from the construction site. To analyze the impact of PM emissions during construction activities and throughout the day, hourly concentrations from 0:00 to 24:00 was recorded as characteristic data to observe PM emissions throughout the day, and 12-hour average PM concentration from 7:00 to 19:00 was recorded as characteristic data for PM emissions during the working period. Based on above data, the emission increments in the two time intervals can be calculated respectively, providing a basis for subsequent research. The average temperature, humidity, atmospheric pressure, and wind speed at above mentioned time point was recorded as the mean of the values observed at the three monitoring points.

#### Monitoring data analysis

1. Correlation analysis

Correlation analysis is a statistical method for determining unknown relationships between two or more variables. The correlation coefficient reflects the strength and direction (negative or positive) or the relationship between the variables. The correlation coefficients most commonly used to analyze relationships between two variables are Pearson’s correlation coefficient and Spearman’s rank correlation coefficient. The Pearson correlation coefficient is suitable for analyzing continuous normally distributed variables and evaluates linear relationships, while Spearman correlation is used in cases involving categorical variables with obviously non-normal or unknown distributions. In this work, Pearson correlation is used to analyze the relationships between PM concentrations at the site and in the city after testing the gathered data being normally distributed. In addition, Spearman correlation is used to analyze the relationships between the PM concentration increments and meteorological factors because the latter were not normally distributed.

(2) Paired sample *t*-test

The paired sample *t*-test evaluates the significance of differences between paired sets of observations. It is generally used to compare normally distributed data with small sample sizes. In this study, it was used to evaluate the significance of differences between upwind and downwind PM concentrations in order to determine whether the construction work significantly increased PM concentrations in the surrounding areas.

(3) Partial least squares (PLS) regression

PLS is an advanced multivariate statistical analysis method. It combines the advantages of principal component analysis and canonical correlation analysis. By decomposing and screening information, the problem of multicollinearity of variables can be effectively solved by generating new completely independent variables. In this way, PLS combines regression analysis with data simplification and reveals correlations between variables. It is especially valuable when dealing with small sample sizes and multicollinear independent variables. In this study, PLS was used to evaluate the influence of individual construction activities and PM reduction measures on PM concentration increments. This was done because PM measurements acquired during individual construction activities or while implementing specific PM reduction measures in a single construction project cannot satisfy the requirements of the conventional Pearson or Spearman methods in terms of data size and multicollinearity.

The indicators, methods and research objects involved in monitoring data analysis in this paper are summarized in Table 2.

**Table 2:** Summary of monitoring data analysis indicators, methods and research objects

No.	Indicators	Method	Research object
1	TSP, PM_10_ and PM2.5 concentrations (12-hour average and 24-hour hourly concentrations)	Pearson correlation analysis	the relationship between the PM concentrations at the site and the averages for Guangzhou
2		Paired sample t-test	significance of differences between upwind and downwind PM concentrations
3		PLS regression	influence of individual construction activities and PM reduction measures on PM concentration increments

## Results and discussion

This section presents the empirical results and their analysis. To begin with, the overall PM concentrations in upwind and downwind of the construction site are analyzed to determine whether the earthwork and foundation construction had a significant impact on atmospheric pollution. In addition, the correlations between PM concentrations and meteorological parameters are evaluated to identify potentially factors influencing PM. Finally, the impacts of individual construction activities and PM reduction measures on the hourly and daily PM concentration increments are analyzed to determine which construction activities generate the highest PM emissions and to identify effective measures to reduce construction PM.

The analysis was based on monitoring data collected on Fridays, Saturdays, and Sundays over the 3-month monitoring period to limit the influence of other industrial activities that are mainly performed on weekdays. Meanwhile, the construction activities of the project did not stop or reduce the workload during the monitoring period (Fridays, Saturdays, and Sundays), so the monitoring results are representative. As a result, PM concentration data representing 31 days was used in the analysis; results for 6 days were excluded due to incomplete monitoring data. All statistical analyses were performed using SPSS26.0 (https://www.ibm.com/products/spss-statistics).

### PM concentration in upwind and downwind directions

**Fig. 4:**
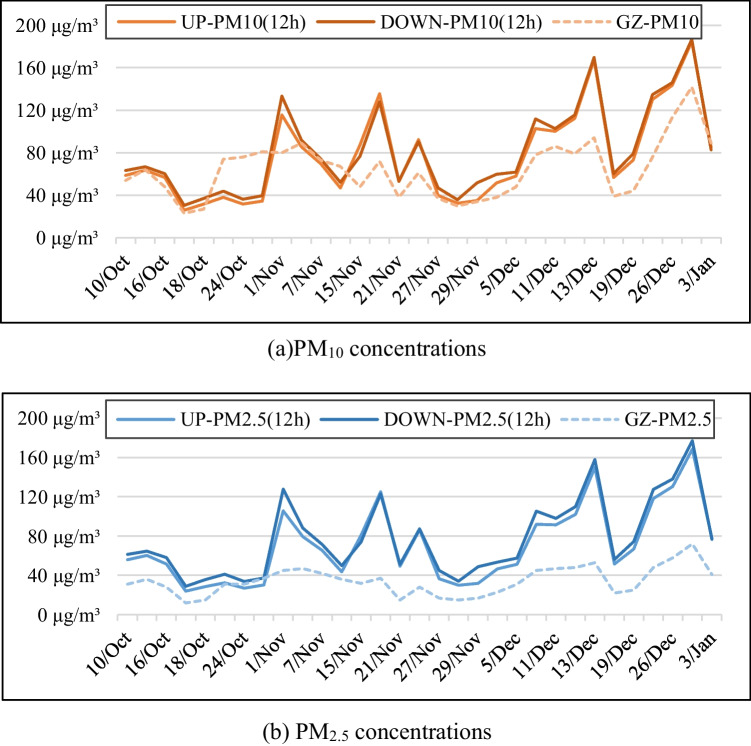
Twelve hour average concentrations of PM_10_ (top) & PM_2.5_ (bottom) and average concentrations of PM_10_ & PM_2.5_ in Guangzhou (GZ) during the study period

The 12-h average concentrations of PM_10_ and PM_2.5_ at the construction site and their daily average concentrations in Guangzhou during the same period are shown in Fig. 4, and the results of a Pearson correlation analysis of the relationship between the PM concentrations at the site and the averages for Guangzhou are shown in Table 3. The TSP concentration is not included in this analysis because daily average TSP concentrations are not officially monitored in China.

**Table 3:** Pearson correlation analysis of PM concentrations at the construction site and the average PM concentrations for Guangzhou at the same time

	Up-wind direction		Down-wind direction	
	PM10	PM2.5	PM10	PM2.5
Correlation	0.763	0.840	0.768	0.851
*P*-value	0.000	0.000	0.000	0.000

During the data collection period, the 12-h average concentration ranges of TSP, PM_10_ and PM_2.5_ in the upwind direction were 30.83 to 221.28 μg/m^3^, 25.92 to 184.6 μg/m^3^, and 24.01 to 168.27 μg/m^3^, respectively. The corresponding ranges in the downwind direction were 36.11 to 223.74 μg/m^3^, 30.32 to 186.72 μg/m^3^, and 28.89 to 176.71 μg/m^3^, respectively. The PM concentrations in both directions thus varied considerably. The full set of collected data is presented in the Appendix.

Figure 4 shows that the 12-h average concentrations of PM_2.5_ and PM_10_ at the construction site exhibited a similar pattern of variation to the background concentrations in Guangzhou. Moreover, as shown in Table 3, the *p*-values for the correlations between the PM concentrations at the construction site and the corresponding averages for Guangzhou were 0.000, indicating that the PM concentrations at the site were very closely related to the background concentrations in the city.

To determine whether the construction work affected PM concentrations in the surrounding area, paired samples *t*-tests were conducted to evaluate the significance of the differences between the average PM concentrations measured at the upwind and downwind monitoring sites. The means and standard deviations of the 12-h upwind and downwind PM concentrations are shown in Table 4 and the results of the paired samples *t*-test for 12- and 24-h PM concentrations in upwind and downwind areas are shown in Table 5. The null hypothesis was that there was no significant difference between the average PM concentrations in the upwind and downwind directions.

**Table 4:** Means and standard deviations of up- and downwind 12- and 24-h PM concentrations

Indicator	Mean (μg/m^3^)	Standard deviation (μg/m^3^)	Indicator	Mean (μg/m^3^)	Standard deviation (μg/m^3^)
UP-TSP(12h)	92.6	50.9	UP-TSP(24h)	96.2	55.8
DOWN-TSP(12h)	97.3	49.7	DOWN-TSP(24h)	99.3	53.2
UP-PM10(12h)	77.4	42.4	UP-PM10(24h)	80.4	46.4
DOWN-PM10(12h)	81.4	41.4	DOWN-PM10(24h)	83.6	44.8
UP-PM2.5(12h)	70.7	38.6	UP-PM2.5(24h)	73.3	42.3
DOWN-PM2.5(12h)	77.2	39.1	DOWN-PM2.5(24h)	79.4	42.3

**Table 5:** Paired samples *t*-test results for upwind and downwind 12- and 24-h PM concentrations

Indicator	*t*	*P*-value
△TSP(12h)	−4.057	0.000
△PM10(12h)	−4.083	0.000
△PM2.5(12h)	−6.969	0.000
△TSP(24h)	−2.703	0.011
△PM10(24h)	−3.536	0.001
△PM2.5(24h)	−7.079	0.000

△ indicates the PM concentration increment, △TSP(12h) = UP-TSP(12h) – DOWN-TSP(12h), and the same hereinafter

Table 4 shows that the 12-hour average concentrations of TSP, PM_10_ and PM_2.5_ in the upwind direction were 92.6±50.9 μg/m^3^, 77.4±42.4 μg/m^3^, and 70.7±38.6 μg/m^3^, and the 24-hour average concentrations were 96.2±55.8 μg/m^3^, 80.4±46.4 μg/m^3^, and 73.3±42.3 μg/m^3^, respectively; the corresponding 12-h averages in the downwind direction were 97.3±49.7 μg/m^3^, 81.4±41.4 μg/m^3^, and 77.2±39.1 μg/m^3^, while the 24-hour average concentrations were 99.3±53.2 μg/m^3^, 83.6±44.8 μg/m^3^, and 79.4±42.3 μg/m^3^, respectively. The average concentrations of all three PM types in the downwind direction were clearly higher than in the upwind direction. The paired samples *t*-test results presented in Table 5 show that the *p*-values for the differences between TSP, PM_10_ and PM_2.5_ in the upwind and downwind directions were below 0.05 and the *t*-values were negative, indicating that the average concentrations in the downwind direction were significantly higher than in the upwind direction. Both descriptive indicators and paired tests thus show that the construction activity significantly increased PM concentrations in the downwind direction. This indicates that it is important to control construction PM emissions and minimize their impact on the surroundings.

### Correlations between construction PM and meteorological factors

The temperature, humidity, atmospheric pressure, and wind speed at the construction site were recorded throughout the study’s duration. Spearman correlation analysis was then used to investigate the correlations between the PM concentration increments and meteorological factors (see Table 6).

**Table 6:** Spearman correlation analysis of the relationships between PM concentration increments and meteorological parameters

Indicator		Temperature	Humidity	Atmospheric pressure	Wind speed
△TSP(12h)	Correlation	−0.165	−.481*	0.272	.472*
	*P*-value	0.375	0.006	0.139	0.007
△PM10(12h)	Correlation	−0.163	−.473*	0.273	.486*
	*P*-value	0.381	0.007	0.137	0.006
△PM2.5(12h)	Correlation	−0.245	−0.107	0.095	0.014
	*P*-value	0.185	0.566	0.612	0.938

* indicates correlations significant at the *p* < 0.01 level (2-tailed)

The *p*-values for the correlations of the PM concentration increments with the temperature and atmospheric pressure were above 0.05, indicating the absence of a statistically significant relationship. Conversely, the corresponding *p*-values for the wind speed and humidity were below 0.05, indicating that these meteorological factors were significantly correlated with the PM concentration increments. The correlation coefficient for wind speed was about 0.5 while that for humidity was around −0.5, indicating that the former had a moderate positive correlation with the TSP and PM_10_ concentration increments while the latter had a moderate negative correlation. These results are in line with our previous research (Yan et al. 2020). No significant correlation was observed between the PM_2.5_ increment and humidity. This suggests that water spraying and the use of fog cannons may effectively reduce PM emissions, particularly those of TSP and PM_10_.

It should be noted that each construction project has unique characteristics including differences in site characteristics and conditions as well as the specific construction activities undertaken. Therefore, while some previous studies have obtained results similar to those reported here (Yan et al. 2020), different relationships between meteorological parameters and PM emissions have been observed in other cases. For example, Yan et al. (2019) found that there was no appreciable correlation between PM concentration increments and any individual meteorological factor, while Fan et al. (2011) found that the PM_10_ concentration correlated positively with wind speed, temperature, and humidity. Further research is thus needed to clarify the reasons for these inconsistencies. However, such work is outside the scope of this study.

### Hourly changes in PM concentration increments

**Fig. 5:**
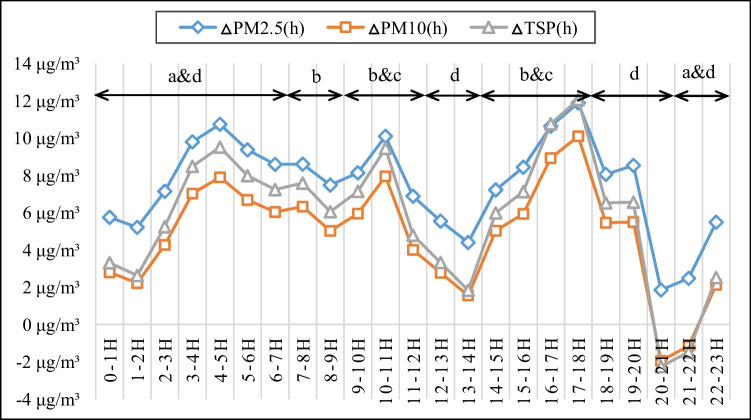
Hourly TSP, PM_10_, and PM_2.5_ concentration increments over 24 hours, plotted values are averages representing 31 monitoring days. (a, b, c and d refer to specific construction activities: a = material transportation and loading-unloading; b = material processing, reinforcement binding, and formwork erection; c = earthwork; d = idling after work)

The hourly average concentration increments for TSP, PM_10_, and PM_2.5_ from 0:00 to 24:00 over the 31 monitoring days of the study are shown in Fig. 5. The hourly concentration increments of all three PM types varied in similar ways over time, rising from 1:00 to 5:00, 8:00 to 11:00, 13:00 to 18:00, and 20:00 to 23:00.

The maximum hourly concentration increments for TSP, PM_10_ and PM_2.5_ were 12.07 μg/m^3^, 10.10 μg/m^3^, and 11.89 μg/m^3^, respectively, and the corresponding minima were -2.26 μg/m^3^, −1.95 μg/m^3^, and 1.85 μg/m^3^, respectively. As shown in Fig. 5, the hourly concentration increments of PM2.5 exceeded those for TSP and PM_10_. The findings of Yan et al. (2020) suggest that this is probably because larger particles with high settlement speeds would have landed on the ground before reaching the monitoring point outside the construction site.

The construction crews entered the site between 7:00 and 9:00. A large number of them, predominantly reinforcement workers and scaffolders, entered between 7:00 and 8:00. The main activities conducted during this period were wood formwork and reinforcement processing, reinforcement binding, and formwork erection, and these activities caused the downwind concentrations of TSP, PM_10_ and PM_2.5_ to increase by 7.59 μg/m^3^, 6.32 μg/m^3^, and 8.60 μg/m^3^, respectively. The PM emissions were mainly attributed to the processing of formwork and reinforcement as well as collisions between building materials and the dusty ground.

From 11:00 to 13:00, workers left the construction site in shifts for their lunch breaks and construction activities were gradually suspended. As a result, the concentration increments of TSP, PM_10_ and PM_2.5_ fell dramatically. During the break time from 13:00 to 14:00, the concentration increments reached their lowest levels over the day (1.55~4.40 μg/m^3^).

From 9:00 to 11:00 and from 14:00 to 18:00, the concentration increments of TSP, PM_10_ and PM_2.5_ increased to 12.07 μg/m^3^, 10.10 μg/m^3^, and 11.89 μg/m^3^, respectively. The main construction activities during these periods were earth excavation and backfilling, formwork erection, and the processing of wood formwork and reinforcement. This was the period when the intensity of the construction work, the number of workers on site, and the quantities of material and equipment in use were all at their highest. During this period, the ground was excavated, rolled, and broken by construction equipment. At the same time, the use of pile-driving machines and transport vehicles cause the ground to vibrate, loosening soil and surface particles, and excavators and transportation vehicles drive repeatedly over the exposed rock and soil, causing friction, shear forces, and skiving forces. As a result, particles are lifted into the air, causing the concentration increments of PM_2.5_, PM_10_ and TSP to increase markedly.

From 18:00 onwards, the working days of the construction workers ended and the concentration increments of PM_2.5_, PM_10_, and TSP gradually declined. The cessation of construction activities allowed suspended particles to gradually settle; as a result, the PM_10_ and TSP concentration increments fell to 0. At 8 p.m., the concentration increments of TSP, PM_10_, and PM_2.5_ reached their lowest levels over the day: −2.26μg/m^3^, −1.95μg/m^3^, and 1.85μg/m^3^, respectively. Some of the PM concentration increments were negative between 20:00 and 22:00 because external sources became the dominant contributors of measured PM rather than the construction site. These external particles are carried by the wind from the upwind direction to the downwind direction, and their movement would be hindered by the enclosure around the construction site; consequently, some of the measured PM concentrations at the upwind measuring point exceeded those at the downwind points. Construction materials were transported to the construction site after 21:00 at night, with frequent vehicle access and loading-unloading of construction materials. As a result, the concentration increments of TSP, PM_10_ and PM_2.5_ increased rapidly to 9.51 μg/m^3^, 7.89 μg/m^3^, and 10.73 μg/m^3^, respectively between 21:00 and 4:00 on the following day. During this period, the concentration increments of TSP and PM_10_ increase more sharply than that of PM_2.5_, presumably because vehicle movement and material loading-unloading cause greater increases in the concentrations of large particles than those of small particles.

The above results indicate that different construction activities generate different levels of PM emissions. It is therefore desirable to decompose the contributions of individual construction activities to the PM concentration increments and identify effective targeted PM reduction measures accordingly.

### Impacts of construction activities and PM reduction measures

The main construction activities conducted during the earthwork and foundation stage were reinforcement binding, foundation formwork erection, concrete pouring, hammer piling, and earthwork operations (excavation and backfilling). PM reduction measures applied in this case during these activities included water spraying and the use of a fog cannon. The relationships between construction activities, reduction measures, and PM concentration increments were evaluated by using PLS. Specifically, the relationships are between the PM concentration increments (the dependent variables, y) and five independent variables: fog cannon use (x1), water spraying (x2), hammer piling (x3), the earthwork volume (x4), and the concrete pouring volume (x5). The results obtained are illustrated in Tables 7 and 8. Table 7 shows the proportion of the total variance explained by five latent variables (t1-t5) generated during the PLS analysis. Table 8 shows the regression coefficients of the independent variables and their variable importance in the projection (VIP) values for each of the five latent variables. The VIP values reflect the degree to which the independent variable explains the dependent variable in relation to the latent variables. Importantly, the VIP reflects both the direct impact of the variable in question on the dependent variable and the indirect impact of other independent variables through the variable in question. It is generally considered that independent variables with VIP values below 0.8 have very limited effects on the dependent variable (Wold 1995).

**Table 7:** Proportion of variance explained by the latent variables T1 to T5

Indicator	Latent variables T	X variance	Cumulative X variance	Y variance	Cumulative Y variance (*R*-squared)	Adjusted *R*-squared
ΔTSP	1	0.305	0.305	0.285	0.285	0.260
	2	0.201	0.506	0.111	0.396	0.353
	3	0.202	0.708	0.022	0.418	0.353
	4	0.172	0.880	0.003	0.420	0.331
	5	0.120	1.000	0.000	0.420	0.304
ΔPM10	1	0.305	0.305	0.285	0.285	0.260
	2	0.201	0.506	0.111	0.396	0.353
	3	0.202	0.708	0.022	0.418	0.353
	4	0.171	0.880	0.003	0.420	0.331
	5	0.120	1.000	0.000	0.420	0.304
ΔPM2.5	1	0.328	0.328	0.281	0.281	0.257
	2	0.184	0.513	0.103	0.385	0.341
	3	0.200	0.713	0.018	0.402	0.336
	4	0.162	0.875	0.002	0.405	0.313
	5	0.125	1.000	0.000	0.405	0.286

**Table 8:** Regression coefficients and variable importance in the projection values for the independent variables in the PLS model

Indicator	X	Regression coefficient	Variable Importance in the Projection				
			t_1_	t_2_	t_3_	t_4_	t_5_
ΔTSP	Constant	−0.243					
	x_1_	0.872	1.025	0.945	0.979	0.976	0.976
	x_2_	−1.163	0.427	1.223	1.204	1.203	1.203
	x_3_	0.753	1.542	1.356	1.341	1.339	1.339
	x_4_	0.391	1.070	0.915	0.941	0.944	0.944
	x_5_	0.038	0.496	0.562	0.584	0.589	0.589
ΔPM10	Constant	−0.241					
	x_1_	0.871	1.021	0.942	0.977	0.974	0.974
	x_2_	−1.165	0.429	1.223	1.204	1.204	1.204
	x_3_	0.752	1.541	1.355	1.340	1.339	1.339
	x_4_	0.391	1.070	0.915	0.941	0.944	0.944
	x_5_	0.036	0.502	0.564	0.586	0.591	0.591
ΔPM2.5	Constant	−0.355					
	x_1_	0.700	0.922	0.819	0.849	0.846	0.846
	x_2_	−0.925	0.082	1.04	1.03	1.031	1.031
	x_3_	0.819	1.538	1.404	1.389	1.387	1.387
	x_4_	0.441	1.277	1.118	1.127	1.127	1.127
	x_5_	0.094	0.383	0.552	0.584	0.590	0.590

Both independent and dependent variables in the table have been standardized. T1, t2, t3, t4, and t5 represent the first five latent factors extracted

Five latent factors were sufficient to explain 100% of the variation in the original independent variables. However, because the PM emissions at the construction site were affected by several different construction activities but only the most common activities were considered in this analysis, the latent variables explained only around 40% of the variation in the dependent variables. These results justify further VIP analysis. As shown in Table 8, the VIP values of the independent variables x1, x2, x3, and x4 were generally above 0.8, indicating that water spraying, fog cannon use, hammer piling, and the earthwork volume all had important effects on the PM concentration increment. Hammer piling had the highest VIP value, indicating that this activity played the most important role in explaining the observed changes in the PM concentration increment. Water spraying affected the TSP and PM_10_ concentration more significantly than that of PM_2.5_. However, the VIP value of x5 was below 0.8, indicating that the concrete pouring volume had little effect on the PM concentration increments during the earthwork and foundation stages. This may be because the ready-mixed commercial concrete used at the site had a high moisture content and was thus unlikely to release significant quantities of airborne PM.

The regression coefficients of x1, x3, and x4 were positive, indicating that hammer piling, earthwork volume, and fog cannon use were associated with higher PM concentration increments. The positive correlation for the fog cannon may be because it was mainly used at times when PM concentrations were expected to be high. However, the spatial extent of the water mist generated by the fog cannon was limited, explaining why the PM concentration increments generally increased when the fog cannon was in use. The regression coefficient of x2 was negative, suggesting that water spraying reduced the PM concentration increments. Additionally, the incremental changes in the TSP and PM_10_ concentrations following a one-unit change in x2 were larger than that for PM_2.5_, indicating that water spraying had a stronger effect on coarse particles than on fine ones (Table 8). Conversely, the incremental increase in PM_2.5_ was smaller than that of PM_10_ and TSP when x1 changed by one unit, suggesting that the fog cannon was more effective at limiting PM_2.5_ emissions than those of TSP and PM_10_. The regression coefficient of x5 was low, indicating that the volume of poured concrete had little effect on the PM concentration increments.

**Fig. 6:**
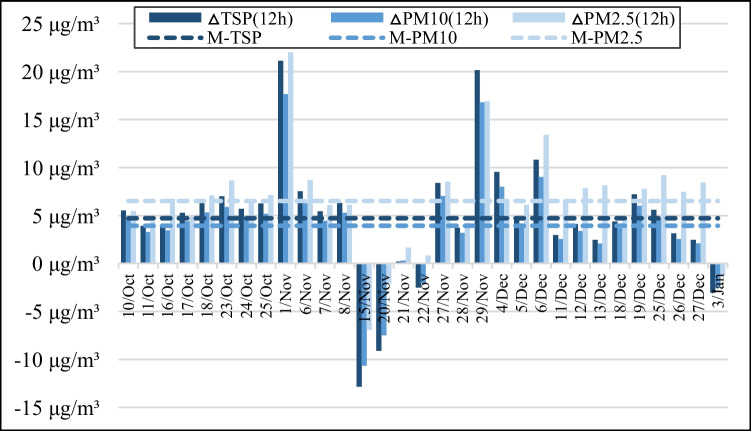
Twelve hour average and overall average PM concentration increments

Figure 6 shows 12-h average PM concentration increments for the 31 monitoring days included in the analysis. These values represent the average concentration increments of TSP, PM_10_ and PM_2.5_ over each full working day. The highest PM concentration increments occurred on November 1^st^, when the average TSP, PM_10_ and PM_2.5_ concentration increments were 21.11 μg/m^3^, 17.65 μg/m^3^, and 22.01 μg/m^3^, respectively. The lowest 12-h averages were observed on November 15^th^ (−12.82 μg/m^3^, −10.63 μg/m^3^, and −6.89 μg/m^3^, respectively). The average 12-h concentration increments of TSP, PM_10_ and PM_2.5_ over the 31 days were 4.7 μg/m^3^, 4.0 μg/m^3^, and 6.5 μg/m^3^, respectively.

The 12-h averages were used to complement the relationships between construction activities and PM emissions during the monitoring period in more detail. From October 17^th^ to October 18^th^, October 23^rd^ to October 25^th^, November 20^th^ to November 22^nd^ and on November 15^th^, the main activities conducted at the site were reinforcement binding, foundation and road formwork erection, concrete pouring, and hammer piling, so the concentration increments of TSP, PM_10_, and PM_2.5_ were very high.

The concentration increments on October 24^th^ were lower than those on October 23^rd^. Additionally, the largest and smallest concentration increments were observed on November 15^th^ and November 21^st^, respectively. These outcomes can be attributed to the use of PM reduction measures: the fog cannon and water spraying were not used on November 15^th^ and October 23^rd^, but both methods were implemented on October 24^th^ and November 21^st^. It thus seems that the implementation of reduction measures can significantly lower the concentration of construction PM in the air.

From November 27^th^ to 29^th^, the PM concentration increments increased markedly. The field researchers found that a large quantity of construction waste awaiting treatment was stacked near POINT-2 in the downwind direction during this period, including wood formwork, reinforcement, and cement bags. As a result, POINT-2 was surrounded and had very poor air circulation, leading to a sharp increase in measured PM concentrations.

On December 6^th^, December 18^th^, and December 19^th^, the volume of excavated earth was high, so the PM concentration increments were also high. As the earthworks were gradually completed, the earthwork area in the foundation pit decreased and the amount of earthwork excavation and backfilling gradually declined. Consequently, the PM concentration increments also fell between the 11^th^ and 13^th^ of December and also between the 25^th^ and the 27^th^.

In sum, the empirical data presented above show that the concentration increment of construction PM is affected by on-site construction activities, the implementation of reduction measures, and meteorological conditions. In addition, because of the effects of PM settlement, it is influenced by the distance between the monitoring point and the pollution source.

### Human health damage from PM emissions

Based on data from 652 cities in 24 countries and regions, Liu et al. (2019) evaluated the associations of PM_10_ and PM_2.5_ with daily respiratory mortality. The results of this study can be used to quantitatively evaluate the health damage of PM emissions on construction workers and surrounding residents. As shown in 4.4, The average 12-hour concentration increments of PM10 and PM2.5 over the 31 days were 4.0 μg/m^3^, and 6.5 μg/m^3^, respectively. Therefore, in the construction site and surrounding areas of the research object, PM_2.5_ emission will cause increases of 0.44% in daily all-cause mortality, 0.36% in daily cardiovascular mortality, and 0.48% in daily respiratory mortality; while the corresponding increase for PM_10_ emission were 0.176%, 0.144%, 0.188%. The calculated results show that the increase of PM concentration caused by construction activities will bring health risks to construction workers and surrounding residents, and active PM reduction measures are essential.

### Summary of results

The results of this long-term empirical study can be summarized as follows:

There were significant differences between upwind and downwind PM concentrations when construction activities were being undertaken. Construction PM thus had a significant impact on the ambient air quality in the downwind area, and this impact decreased sharply when the construction work ended.

The concentration increments of TSP and PM_10_ correlated negatively with humidity and positively with wind speed. However, no significant correlations were found between PM concentration increments and temperature or atmospheric pressure during the data collection period.

The main construction activities associated with increased PM emissions during the earthwork and foundation stage were hammer piling, earthwork, waste stacking, and materials transportation. However, PM emissions from concrete pouring were negligible. Water spraying was found to be an effective measure for reducing TSP and PM_10_ emissions, while the use of a fog cannon effectively reduced PM_2.5_ emissions. However, fog cannons should be seen as auxiliary tools for reducing PM emissions because they are only effective in a small area within their spraying radius.

Based on obtained characteristics of PM emissions from construction sites, the following recommendations for PM reduction are proposed: (1) The monitoring data shows that, among all kinds of PM concentration increment from emission sources of construction activities, concentration of PM_2.5_ increased dramatically, so the more focus should be paid on PM_2.5_ reduction and control; (2) The correlation analysis between air humidity and dust particle concentration is negative. So the methods of road sprinkling, fog cannon and fence spraying are able to control the transmission, and ultimately reduce PM emissions; (3) The correlation analysis between wind speed and dust particle concentration increment shows that the greater the wind speed, the greater the downwind dust concentration increment. Therefore, changing the wind speed at the construction site by adjusting the height of the construction fence is also one of the strategies for the control of transmission route and PM emission; (4) This study identifies construction activities with high PM emission sources such as piling and earthwork, the construction worker as receptor should enhance pollution protection for receptors involved in such construction activities.

## Conclusion

PM emissions from a real construction project were monitored during the earthwork and foundation stages by measuring the concentrations of TSP, PM_10_ and PM_2.5_ upwind and downwind of the construction site. In addition, the weather conditions during the measurement period were recorded together with the construction activities that were performed and the applied PM reduction measures. The gathered data were subjected to quantitative statistical analysis, providing robust insights into construction PM emissions during the earthwork and foundation stages and the factors influencing them. The results obtained will be useful to site managers seeking effective measures for reducing construction PM emissions.

The gathered data showed that construction work during the foundation and earthwork stages significantly affected air quality in surrounding areas. In addition, there was a clear correlation between measured PM emissions and humidity, supporting the effectiveness of measures such as water spraying, the use of fog cannons, and ground and tire watering to reduce PM emissions. The correlation between humidity and PM emissions also means that construction managers can predict future PM emissions based on rainfall and humidity forecasts, allowing them to decide in advance when PM reduction measures should be applied.

This empirical case also identified the main construction activities with considerable PM emission, as well as effective PM reduction measures under different conditions. Decision makers at construction sites can choose suitable PM reduction measures according to these construction activities. For example, the results obtained suggest that water spraying systems should be activated (with fog cannons being used as auxiliary tools) to limit emissions of TSP, PM_10_ and PM_2.5_ during earthwork operations and hammer piling. Control measures may also be warranted during transportation of construction materials and waste stacking.

This work contributes to the field by presenting and analyzing empirical data on the characteristics of PM emissions during the earthwork and foundation construction stages of a major construction project. However, some areas where further research is needed stand out. First, the monitoring instruments in this study were distributed at the boundary of the site because the study’s objective was to evaluate the impact of construction PM emissions on the surrounding area. In future, it would be desirable to directly measure the PM emissions of individual construction activities and evaluate their impact on workers in the construction site. In addition, future studies can examine a wider range of construction activities and PM reduction measures.

## Data Availability

Not applicable.

## Appendix. Average concentrations and increments of PM in the upwind and downwind directions during monitoring

**Table:**

Date	TSP			PM10			PM2.5		
	Upwind	Down-wind	Increment	Upwind	Down-wind	Increment	Upwind	Down-wind	Increment
10-Oct	70.2	75.7	5.5	58.7	63.3	4.6	55.9	61.3	5.4
11-Oct	75.8	79.7	3.9	63.4	66.7	3.3	60.3	64.7	4.4
16-Oct	68.0	72.0	4.0	56.8	60.3	3.5	51.5	58.3	6.7
17-Oct	30.8	36.1	5.3	25.9	30.3	4.4	24.0	28.9	4.9
18-Oct	37.8	44.1	6.3	31.7	37.0	5.3	28.6	35.7	7.1
23-Oct	45.2	52.2	7.0	37.9	43.8	5.9	32.6	41.2	8.7
24-Oct	37.8	43.4	5.7	31.7	36.4	4.7	27.1	33.9	6.7
25-Oct	41.1	47.3	6.3	34.5	39.6	5.2	30.2	37.3	7.1
1-Nov	138.5	159.6	21.1	115.6	133.2	17.7	105.7	127.7	22.0
6-Nov	102.4	110.0	7.5	85.5	91.8	6.3	79.5	88.2	8.7
7-Nov	83.3	88.8	5.4	69.7	74.1	4.4	65.5	71.6	6.1
8-Nov	56.1	62.4	6.3	47.0	52.3	5.3	43.7	49.9	6.1
15-Nov	104.4	91.6	-12.8	87.3	76.6	-10.6	80.9	74.0	-6.9
20-Nov	162.5	153.4	-9.1	135.6	128.1	-7.5	125.0	122.9	-2.1
21-Nov	63.3	63.5	0.2	52.9	53.2	0.3	49.5	51.2	1.7
22-Nov	110.6	108.1	-2.5	92.4	90.3	-2.1	86.5	87.3	0.8
27-Nov	47.4	55.8	8.4	39.7	46.7	7.0	36.5	45.0	8.5
28-Nov	38.8	42.5	3.7	32.5	35.7	3.2	30.0	34.3	4.2
29-Nov	41.6	61.8	20.1	34.9	51.7	16.8	32.0	48.9	16.9
4-Dec	61.7	71.2	9.5	51.7	59.7	8.0	46.8	53.4	6.7
5-Dec	69.4	73.9	4.5	58.1	61.8	3.8	51.4	57.5	6.1
6-Dec	122.9	133.7	10.8	102.7	111.7	9.0	92.1	105.5	13.4
11-Dec	119.9	122.8	2.9	100.1	102.7	2.5	91.3	97.9	6.6
12-Dec	134.5	138.6	4.1	112.3	115.7	3.4	102.0	109.9	7.8
13-Dec	200.9	203.4	2.5	167.7	169.8	2.1	149.7	157.8	8.1
18-Dec	67.7	72.1	4.4	56.7	60.4	3.7	51.5	56.2	4.7
19-Dec	87.5	94.7	7.2	73.2	79.2	6.0	66.8	74.6	7.8
25-Dec	155.9	161.5	5.6	130.2	134.8	4.7	118.2	127.4	9.2
26-Dec	171.8	174.9	3.1	143.5	146.0	2.5	130.6	138.0	7.5
27-Dec	221.3	223.7	2.5	184.6	186.7	2.1	168.3	176.7	8.4
3-Jan	101.9	98.9	-3.0	85.2	82.7	-2.5	77.9	76.6	-1.2

## References

[CR1] Araújo I, Costa D, Moraes RD (2014). Identification and characterization of particulate matter concentrations at construction jobsites [J]. Sustainability.

[CR2] Azarmi F, Kumar P (2016). Ambient exposure to coarse and fine particle emissions from building demolition. Atmos Environ.

[CR3] Cowherd C, Axetell K, Guenther CM, Jutze GA (1974). Development of emission factors for fugitive dust sources.

[CR4] Dai L, Zanobetti A, Koutrakis P, Schwartz JD (2014). Associations of fine particulate matter species with mortality in the united states: A multicity Time-Series analysis. Environ Health Persp.

[CR5] Dockery D (1994). Acute respiratory effects of particulate air pollution. Annu Rev Publ Health.

[CR6] Faber P, Drewnick F, Borrmann S (2015). Aerosol particle and trace gas emissions from earthworks, road construction, and asphalt paving in Germany: Emission factors and influence on local air quality. Atmos Environ.

[CR7] Fan SB, Li G, Tian G (2011). Fugitive dust emission characteristics from construction site by field measure. Environ Sci Technol.

[CR8] Fan WB, Chen JH, Tang BY, Feng XQ, Sun HL, Zhang Y, Wang J, Jin CY, Luo LH, Jiang T, Wu K, Sun S, Tao J, Qian J, Zheng L (2020). Dust emission characteristics of construction activities in Chengdu. China Environ Sci.

[CR9] Fitz DR, Bumiller K (2000). Evaluation of watering to control dust in high winds. J Air Waste Manage.

[CR10] Fujitani Y, Kumar P, Tamura K, Fushimi A, Hasegawa S, Takahashi K (2012). Seasonal differences of the atmospheric particle size distribution in a metropolitan area in Japan. Sci Total Environ.

[CR11] Ge HY (2018). Characteristics of particulate matter concentrations and their relationship with meteorological factors in turpan. Desert Oasis Meteorol.

[CR12] Guo YH, Wang ZF, Kang H, Zhang XX, Ji Y, Li J, Chen HS (2014). Lnfluence of monitoring index TSP and PM10 on ambient air quality grade at cities of Xinjiang, China. Arid Land Geography.

[CR13] Guttikunda SK, Calori G (2013). A GIS based emissions inventory at 1 km × 1 km spatial resolution for air pollution analysis in Delhi, India. Atmos Environ.

[CR14] Huang LK, Wang K, Wang GZ, Ren Q, Li JS (2014). Analysis of the correlation of TSP, PM10 and PM2.5 in harbin atmosphere. Chem Adh.

[CR15] Lee C, Tang L, Chang C (2001). Modeling of fugitive dust emission for construction sand and gravel processing plant. Environ Sci Technol.

[CR16] Lin FX, Su H, Chen Q, Hu HY, Liang JB (2018). Research on the emission characteristics of dust in building construction dust of cities in pearl river delta region. Guangdong ArchitectureCivil Eng.

[CR17] Liu C, Chen R, Sera F (2019). Ambient particulate air pollution and daily mortality in 652 cities. New England J Med.

[CR18] Luo ZM, Wang DF Ding XH (2022) Accuracy analysis on evaluation indexes of dust suppressant effect. China Safety Sci J 32(1),195-200. (in Chinese) 10.16265/j.cnki.issn1003-3033.2022.01.026

[CR19] Ministry of Labor of the People’s Republic of China. ed. (1996). Method of Measuring Concentration of Dust in the Air on the Principle of Scattered light, Beijing.

[CR20] Moraes RJ, Costa DB, Araujo I (2016). Particulate Matter Concentration from Construction Sites: Concrete and Masonry Works. J Environ Eng.

[CR21] Ngoc L, Park D, Lee Y, Lee Y (2017). Systematic review and Meta-Analysis of human skin diseases due to particulate matter. Int J Env Res Pub He.

[CR22] Raile MM (1996). Characterization of Mud/Dirt Carryout onto Paved Roads from Construction and Demolition Activities.

[CR23] Ruan SL, Jin Y, Li LY, Jing Y (2019). Simulating analysis of point source dust diffusion characteristics in construction sites. Environ Sci Technol.

[CR24] Tian G, Li G, Yan BL, Huang YH, Qin JP (2008). Spatial Dispersion Laws of Fugitive Dust from Construction Sites. Environ Sci.

[CR25] Tian G, Li G, Qin JP, Fan SB, Huang YH, Nie L (2009). Influence of Traffic Restriction on Road and Construction Fugitive Dust. Environmental. Science.

[CR26] USEPA (ed) (1996) Air quality criteria for particulate matter. DC, USA, Washington, Available online: https://cfpub.epa.gov/si/si_public_record_report.cfm?Lab=NCEA&dirEntryId=87903/. Accessed 19 Mar 2023

[CR27] USEPA (ed) (2013) National ambient air quality standards for particulate matter. DC, USA, Washington, Available online: https://www.epa.gov/pm-pollution/national-ambient-air-quality-standards-naaqs-pm/. Accessed 19 Mar 2023

[CR28] Wold S (1995) PLS for multivariate linear modeling. In: H. Van de Waterbeemd (ed) Chemometric methods in molecular design: 195-218.

[CR29] for Europe., World Health Organization Office (2006). Air quality guidelines.

[CR30] Xue Y, Zhou Z, Huang Y, Wang K, Nie T, Nie L, Qin J (2017). Fugitive Dust Emission Characteristics from Building Construction Sites of Beijing. Environmental. Science.

[CR31] Yan H, Ding GL, Li H, Wang YS, Zhang L, Shen G, Feng KL (2019). Field Evaluation of the Dust Impacts from Construction Sites on Surrounding Areas: A City Case Study in China. Sustainability-Basel.

[CR32] Yan H, Ding GL, Feng KL, Zhang L, Li HY, Wang YS, Wu TY (2020). Systematic evaluation framework and empirical study of the impacts of building construction dust on the surrounding environment. J Clean Prod.

[CR33] Yan H, Lu X, Lian Q, Feng KL (2022). The Pollution Characteristics of Dust in the Key Surrounding Area of Construction Site: A Case of Construction Site in Guangzhou. Journal of. Eng Manag.

[CR34] Zhang GQ, Liu ZC, Hoeflinger W, Mauschitz G (2008). Emission characteristics of fugitive dust generation of falling material and its model. J Shandong Agric Univ.

[CR35] Zhao P, Li ZF, Deng RY (2021). Numerical simulation of dust diffusion characteristics during earthwork construction [J]. J Saf Environ.

[CR36] Zhao PS, Feng YC, Zhang YF, Zhu T, Jin J, Zhang XL (2009). Research and application of quantitative model of construction dust emission factor [J]. China Environ Sci.

[CR37] Zhao Y, Yu L, Zhang CH, Wang Q, Jing T, Chen Z (2010). Spatial dispersion laws of particulate matter from construction work site of municipal engineering. Ecol Environ Sci.

[CR38] Zhong LP, Cao Y, Li YW, Pan WP, Xie KC (2010). Effect of the existing air pollutant control devices on mercury emission in coal-fired power plants. J Fuel Chem Technol.

[CR39] Zou K (2021). Application of complete set of on-line dust monitoring technology in construction dust supervision. Brick-Tile.

